# Correlation Between CHA_2_DS_2_-VASc Score and Left Atrial Size in Patients With Atrial Fibrillation: A More Than 15-Year Prospective Follow-Up Study

**DOI:** 10.3389/fcvm.2021.653405

**Published:** 2021-06-28

**Authors:** Chin-Feng Tsai, Pang-Shuo Huang, Jien-Jiun Chen, Sheng-Nan Chang, Fu-Chun Chiu, Ting-Tse Lin, Ling-Ping Lai, Juey-Jen Hwang, Chia-Ti Tsai

**Affiliations:** ^1^Division of Cardiology, Department of Internal Medicine, School of Medicine, Chung Shan Medical University Hospital, Chung Shan Medical University, Taichung, Taiwan; ^2^Division of Cardiology, Department of Internal Medicine, National Taiwan University College of Medicine and Hospital Yun-Lin Branch, Douliu, Taiwan; ^3^Division of Cardiology, Department of Internal Medicine, National Taiwan University College of Medicine and Hospital Hsin-Chu Branch, Hsinchu, Taiwan; ^4^Division of Cardiology, Department of Internal Medicine, National Taiwan University College of Medicine and Hospital, Taipei, Taiwan

**Keywords:** left atrial size, CHA_2_DS_2_-VASc score, stroke, atrial fibrillation, Asia

## Abstract

**Background:** Left atrial (LA) size represents atrial fibrillation (AF) burden and has been shown to be a predictor for AF stroke. The CHA_2_DS_2_-VASc score is also a well-established predictor of AF stroke. It is unknown to cardiologists whether these two risk scores are correlated, whether both are independent prognostic predictors and complimentary to each other, or whether one of them is a major determinant of stroke risk for AF patients.

**Method:** A total of 708 patients from the National Taiwan University Atrial Fibrillation Registry were longitudinally followed up for more than 15 years. Left atrial size was measured by M mode of echocardiography. Adverse thromboembolic endpoints during follow-up were defined as ischemic stroke or transient ischemic attack.

**Results:** The mean age was 72.1 ± 12.9 years, with 53% men. Both LA size and CHA_2_DS_2_-VASc score were associated with the risk of stroke in univariate analyses. There was a weak but significant positive correlation between LA size and CHA_2_DS_2_-VASc score (*r* = 0.17, *P* < 0.0001). Patients with higher CHA_2_DS_2_-VASc scores had a higher mean LA size (*P* < 0.01 for trend). When combining LA size and CHA_2_DS_2_-VASc score in the multivariable Cox model, only CHA_2_DS_2_-VASc score remained statistically significant [HR 1.39 (1.20–1.63); *P* < 0.001].

**Conclusion:** LA size is not an independent predictor of AF stroke, and calculation of CHA_2_DS_2_-VASc score may be an alternative to measurement of echocardiographic LA size when evaluating the risk of stroke for AF patients.

## Introduction

The left atrium (LA) of human heart functions to act as a contractile pump that delivers 15 to 30% of the left ventricular (LV) filling, as a reservoir that collects pulmonary venous return during LV systole, and as a conduit for the passage of stored blood from the LA to the LV during early LV diastole (1). LA enlargement has been proven to be associated with increased cardiovascular events, such as stroke, congestive heart failure, cardiovascular death, and atrial fibrillation (AF) (2). Patients with enlarged LA are more prone to AF, and an enlarged LA is more likely to maintain AF. On the other hand, AF has also been known to affect LA remodeling and geometry. Regardless of whether LA enlargement is a cause for or a consequence of AF, LA structure, and function represent AF burden and duration, conferring a risk for stroke and systemic embolization (3).

Several studies have investigated the relationship between the LA size and stroke in general populations, showing increased risk of stroke when LA was enlarged (4–11). Given that LA enlargement represents increased burden and duration of AF, it is proposed as an independent predictor of risk for stroke among AF populations (11). Therefore, the more the AF burden is, the higher the risk of cardiac embolic stroke is. However, performing echocardiography is an expensive modality and is less feasible in large population screening. Instead, the CHA_2_DS_2_-VASc score has been demonstrated to be another good scoring scheme to predict stroke risk in patients with AF and could be easily calculated and used in routine clinical practice (12). The CHA_2_DS_2_-VASc score is based on a point system in which two points are assigned for a history of stroke or transient ischemic attack (TIA) and age more than 75 years, and one point is assigned for presence of hypertension, diabetes, congestive heart failure, and vascular diseases or a female gender.

Therefore, both LA size and CHA_2_DS_2_-VASc score have been proposed as the predictors of AF-related stroke. However, echocardiography may not be immediately available in all clinics. Calculation of CHA_2_DS_2_-VASc score is an easier tool to use in clinics to predict stroke risk for patients with AF. The aims of this study were to assess whether LA size and the CHA_2_DS_2_-VASc score were correlated and to determine which one was the independent predictor of AF-related stroke in a long prospective AF follow-up cohort in Taiwan.

## Methods

### Patient Population and Follow-Up

The National Taiwan University Atrial Fibrillation Registry (NTUAFR) was first established in Jan 1998, and so far the patients had been followed up for more than 15 years. The NTUAFR includes data on inpatients and outpatients with incident AF. The enrollment was staggered, and details of selection of AF patients have been described previously and according with current guidelines (13–16). The presence of AF was determined by taking the patient's history, serial ECG, and/or ambulatory ECG monitoring. A total of 1,233 patients were recruited. Patients with palpitations without ECG documentation were excluded from the registry (16). Patients with hyperthyroidism were excluded because AF in these patients might be curable. Patients who refused to join the study or were lost to follow-up were also excluded. Finally, the study population consisted of 708 consecutive adult AF patients from the NTUAFR with detailed echocardiographic data. The mean age was 72.1 ± 12.9 years, and 53% (375/708) were men. The study protocols were reviewed and approved by the institutional review committee, and all patients agreed to participate in the study.

### Clinical and Outcome Assessments

At enrollment, the medical history was recorded, and transthoracic echocardiographic data of left atrial and left ventricular dimensions, left ventricular ejection fraction, and severity of valvular heart disease were also recorded. The LA size was determined as the M-mode measurement of the anteroposterior diameter of LA in parasternal long-axis view. We defined the cutoff value of LA enlargement as LA diameter more than 50 mm, as LA diameter more than 50 mm had been previously reported to be associated with a higher risk of stroke and systemic embolization in patients with AF (11). Besides, in our study the LA size was not controlled for body size because it did not significantly affect the results and it has been reported that LA size not controlled for body size was associated with an increased risk of stroke in patients with AF (17).

All the baseline characteristics were collected as previously described (13–15). The medical diagnoses of hypertension (blood pressure consistently above 140/90 mmHg or treated with hypertension medication), heart failure (admission for congestive heart failure), diabetes mellitus (DM) (fasting glucose >125 mg/dL or treatment with oral hypoglycemic agent and/or insulin), and vascular disease (peripheral arterial disease, coronary artery disease, and myocardial infarction) are also searched in the medical records (12).

The CHA_2_DS_2_-VASc scores were calculated and recorded as measures of stroke risk. The CHA_2_DS_2_-VASc score is based on a point system in which two points are assigned for a history of stroke or TIA and age more than 75 years, and one point is assigned for presence of hypertension, diabetes, congestive heart failure, and vascular diseases or a female gender (12). We categorized the patients into 3 groups (CHA_2_DS_2_-VASc score ≦2, 3–5 and ≥6), because CHA_2_DS_2_-VASc score ≦2 is associated with a lower risk of stroke but CHA_2_DS_2_-VASc score ≥6 is associated with a significantly higher risk of stroke (16, 18, 19).

Adverse thromboembolic endpoints during follow-up were defined as incident episodes of ischemic stroke or TIA. Ischemic stroke was defined as sudden-onset and focal or global neurological deficits that were not explained by other origins, with supporting evidence from imaging studies. Hemorrhagic stroke was excluded from the study because it was generally a complication of use of anticoagulant and not related to AF *per se*.

### Statistical Methods

Continuous variables are presented as means ± standard deviation and categorical variables as percentages. The comparison of data was performed by using the chi-squared test (categorical variables) or the Student's *t*-test (continuous variables). The time to first episode of stroke event were depicted with the Kaplan–Meier estimate of the survival function. Difference between the survival curves was tested by the log-rank statistics. The independent effect of variables to predict thromboembolic events was calculated using a Cox proportional hazards regression model. Hazard ratios (HRs) and 95% confidence intervals (CIs) were calculated accordingly. To test the accuracy of different risk factors in predicting the occurrence of thromboembolic events, the receiver operator characteristic curves (ROC) were constructed.

The ROC analyses gave an estimate of the overall discriminate ability of risk predictor by the area under the curve (AUC) statistic or *c* statistic. We calculated sensitivity and specificity across the range of possible cutoff values of risk predictor scores. AUCs and associated 95% CIs were calculated. The correlation between CHA_2_DS_2_-VASc score and LA size was determined by calculating the Pearson's correlation coefficient (*r*). The Cochran–Armitage trend test was used for the trend of increase of LA size with different groups with increasing mean CHA_2_DS_2_-VASc score. *P* < 0.05 was considered statistically significant. Statistical analysis was performed using STATA version 7.0 for Windows (STATA, Inc., Texas, USA).

## Results

### Baseline Characteristics

Baseline characteristics of study patients are summarized in Table 1. The mean age was 72.1 ± 12.9 years, and 53% (375/708) were men. Among the study subjects, 44.9% (318/708) aged over 75 years, 59.9% (424/708) had history of hypertension, 25.8% (183/708) had history of DM, 10.6% (75/708) had history of congestive heart failure, and 32.6% (231/708) had vascular diseases. The mean CHADS_2_-VASc score was 3.02 ± 1.53.

**Table 1:** Baseline characteristics of the patients at enrollment (*n* = 708).

Age, y	72.1 ± 12.9
Age ≧ 75 yrs	44.9% (318/708)
Male	53.0% (375/708)
Congestive heart failure	10.6% (75/708)
Diabetes mellitus	25.8% (183/708)
Hypertension	59.9% (424/708)
Current smoking	27.0% (191/708)
Vascular diseases (CAD or AMI or PAOD)	32.6% (231/708)
CHADS_2_-VASc score	3.02 ± 1.53
Echocardiographic parameters	
Left atrial dimension, mm	43.8 ± 9.4
LVEDD, mm	58.9 ± 16.5
LVESD, mm	33.7 ± 11.9
LVEF, %	64.1 ± 12.3
LV mass, g	245 ± 84

*AMI, acute myocardial infarction; CAD, coronary artery disease; LVEDD, left ventricular end diastolic dimension; LVESD, left ventricular end systolic dimension; LVEF, left ventricular ejection fraction; PAOD, peripheral arterial occlusive disease*.

Regarding the echocardiographic data, 21.3% of the study subjects had LA dimension more than 50 mm. The mean left ventricular ejection fraction was 64.1 ± 12.7 %. The mean LV end-systolic dimension (LVESD) was 58.9 ± 16.5 mm. The mean LV end-diastolic dimension (LVEDD) was 33.7 ± 11.9 mm.

### CHA_2_DS_2_-VASc Score as a Predictor of Incident Stroke Events

At the end of the follow-up, 70 AF patients developed ischemic stroke or TIA. We first investigate whether CHA_2_DS_2_-VASc score was associated with risk of stroke in our cohort (8, 9). As expected, CHA_2_DS_2_-VASc score was incrementally associated with the risk of stroke. Using the univariate Cox model, we found that there was a 42% increase of risk of stroke with a one-point increase of CHA_2_DS_2_-VASc score in this very long follow-up cohort (HR 1.42, 95% CI 1.22–1.66, *P* < 0.001).

**Figure 1:**
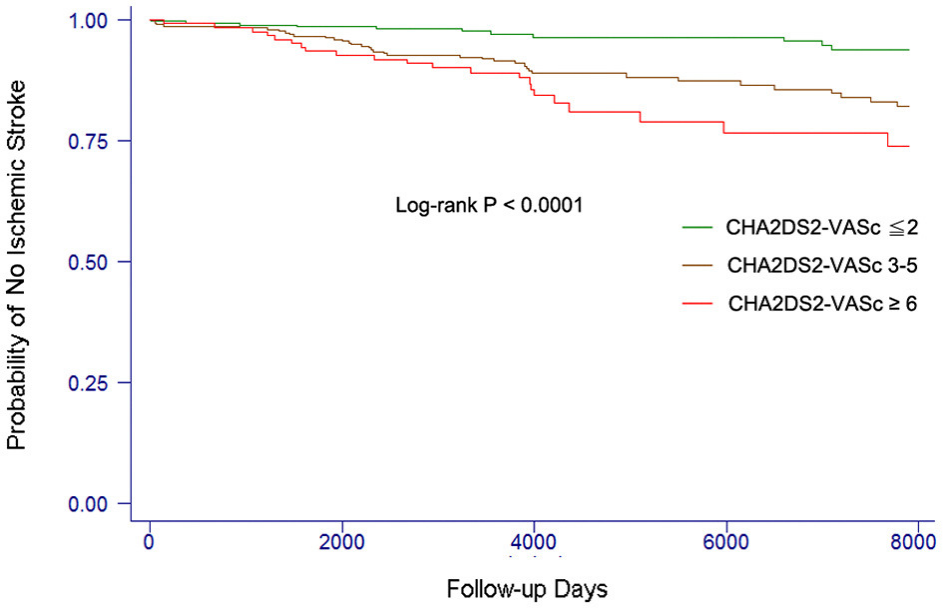
Kaplan–Meier curves showing the development of ischemic stroke among patients with different levels of CHA_2_DS_2_-VASc score. The log-rank analysis showed significant difference (*P* < 0.001).

The event-free survival from stroke comparing patients with different levels of CHA_2_DS_2_-VASc score is shown in Figure 1. The cut-point CHA_2_DS_2_-VASc score for each category was defined to make the case number similar in each group. Patients with a higher CHA_2_DS_2_-VASc score were more likely to develop ischemic stroke or TIA than those with lower CHA_2_DS_2_-VASC score (log-rank *P* < 0.0001).

### Increase of Incident Stroke Event Risk When LA Size Increases

**Figure 2:**
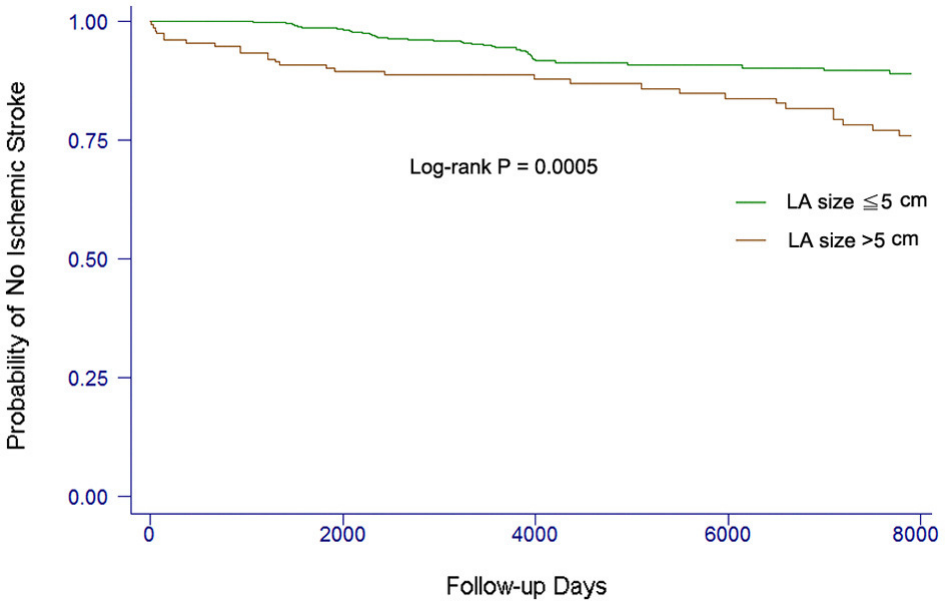
Kaplan–Meier curves showing the development of ischemic stroke among patients with different LA sizes. The log-rank analysis showed significant difference (*P* < 0.001).

In the present study, we also investigated whether the risk of ischemic stroke or TIA was increased when the LA size increased (3, 8, 9, 11). We found that LA size was incrementally associated with the risk of stroke. Using the univariate Cox model, there was a 30% increase of risk of stroke with a 1-mm increase of LA size (HR 1.30, 95% CI 1.04–1.62, *P* = 0.019). The event-free survival from stroke comparing patients with different LA size is shown in Figure 2. Patients with LA size >50 mm were more likely to develop ischemic stroke or TIA than those with LA size ≦50 mm (log-rank *P* = 0.0005).

**Figure 3:**
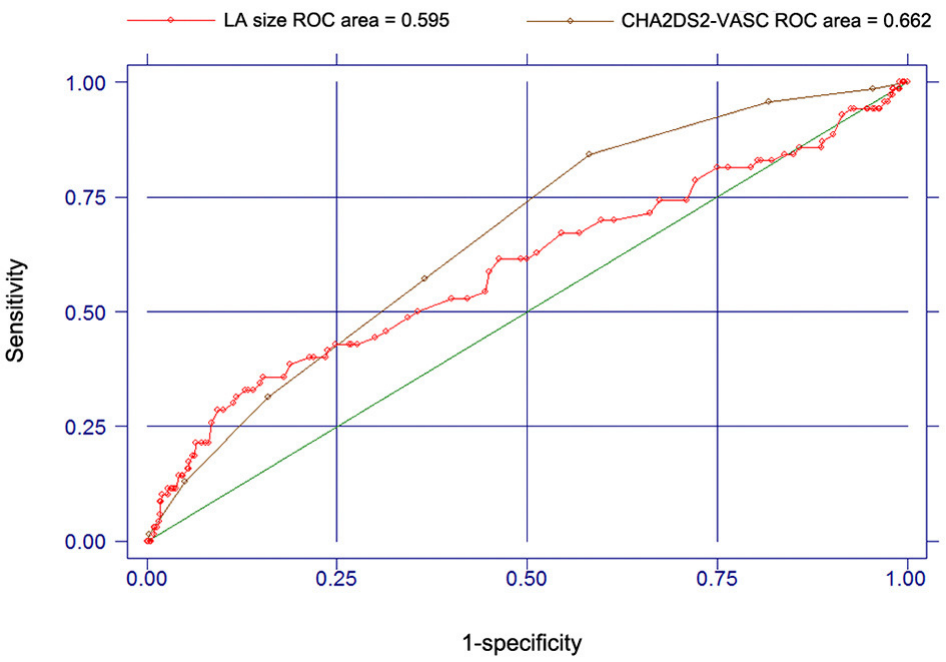
Receiver operating characteristic curves and c-statistics for CHA_2_DS_2_-VASC score and LA size in predicting the occurrence of stroke or TIA.

Since both CHA_2_DS_2_-VASC score and LA size were associated with the risk of stroke for patients with AF, we also use ROC and *c* statistic (AUC statistic) to test the accuracy of CHA_2_DS_2_-VASC score and LA size in predicting the occurrence of stroke or TIA (Figure 3). For the CHA_2_DS_2_-VASC score, the AUC or *c* statistic for stroke events was 0.662 (95% CI, 0.601–0.723). For the LA size, the AUC or *c* statistic for stroke events was 0.595 (95% CI, 0.516–0.674). The *c* statistic of CHA_2_DS_2_-VASC score was larger than that of LA size, but not statistically significant (*P* = 0.187; Figure 3).

### Correlation Between LA Size and CHADS_2_ VASC Score

In the present study we showed both CHA_2_DS_2_-VASc score and LA size could predict the risk of stroke for patients with AF (20, 21). We further hypothesized that the CHA_2_DS_2_-VASc score predicted the risk of stroke through its association with LA size. To address this issue, we first investigated whether CHA_2_DS_2_-VASc score correlated with LA size, which had never been addressed before our study.

**Figure 4:**
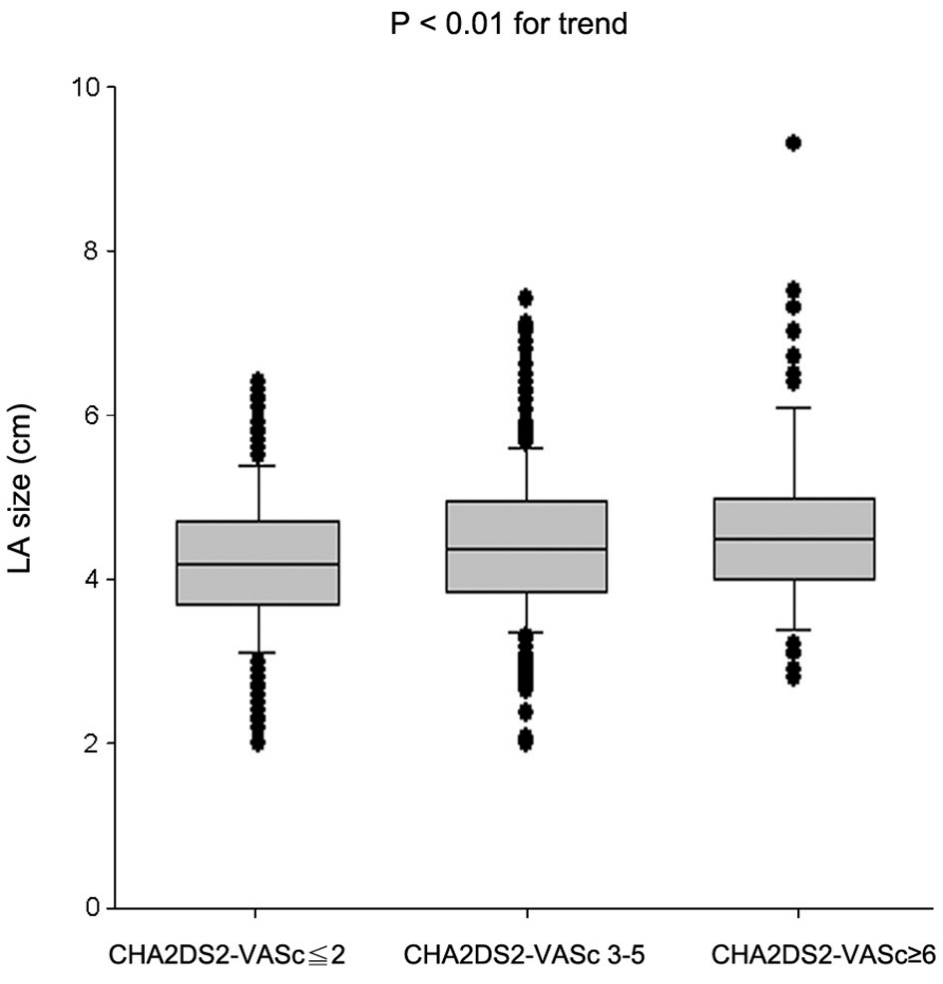
Mean left atrial size for different levels of CHA_2_DS_2_-VASc score.

Interestingly, we found a positive correlation between LA size and CHA_2_DS_2_-VASc score (*r* = 0.17, *P* < 0.0001). There is an increase of mean LA size when the mean CHA_2_DS_2_-VASc score increases (*P* < 0.01 for trend; Figure 4). It indicates that AF patients with a higher CHA_2_DS_2_-VASc score may have a larger LA size.

### The Role of CHA_2_DS_2_-VASc Score and LA Size in Determining the Stroke Risk for Patients With AF

We have shown a positive correlation between the CHA_2_DS_2_-VASc score and LA size, and both CHA_2_DS_2_-VASc score and LA size are predictors of stroke risk for patients with AF. It is logical to speculate that the CHA_2_DS_2_-VASc score predicts the risk of stroke through its association with LA size since LA size represents AF burden. To determine if CHA_2_DS_2_-VASc score predicted the risk of stroke through its association with LA size or if CHA_2_DS_2_-VASc score and LA size are independent predictors, we ran a multivariable Cox model.

Unexpectedly, when both CHA_2_DS_2_-VASc score and LA size were incorporated simultaneously into the Cox model, only CHA_2_DS_2_-VASc score remained statistically significant (HR 1.39; 95% CI 1.20–1.63; *P* < 0.001; Table 2).

**Table 2:** Univariate and multivariable analyses in Cox models.

**Mode of analysis**	**Hazard ration (95% confidence interval)**	***P*-value**
Univariate analysis^*^		
CHADS_2_-VASc score	1.42 (1.22–1.66)	<0.001
Left atrial size	1.30 (1.04–1.62)	0.019
Multivariable analysis^*^		
CHADS_2_-VASc score	1.39 (1.20–1.63)	<0.001
Left atrial size	1.20 (0.96–1.48)	0.106

* *In univariate analyses, single covariate, such as CHADS_2_-VASc score or left atrial size was put into the Cox model. In the multivariate analysis, both CHADS_2_-VASc score and left atrial size were put into the Cox model*.

## Discussion

The present study showed that LA enlargement was significantly associated with an increased risk of stroke in a very long-term AF follow-up cohort, but the association disappeared if the CHA_2_DS_2_-VASc score was also included in the multivariable model. The LA size was positively correlated with the CHA_2_DS_2_-VASc score. These results indicate that the association of LA size with incident stroke in patients with AF is through its correlation with the CHA_2_DS_2_-VASc score and in evaluating the risk of stroke for patients with AF, calculating the CHA_2_DS_2_-VASc score is enough without the need for LA size measurement.

### LA Enlargement, AF Burden, and Risk of Stroke

A recent large-scale, prospective study with 2-year follow-up has shown that LA enlargement is independently associated with an increased risk of stroke and systemic embolization (11). However, another substudy of Atrial Fibrillation Follow-Up Investigation of Rhythm Management (AFFIRM) Study with a 3.5-year follow-up has shown that larger LA size is not associated with increased risk of stroke (22). Finally, sub-analyses of three clinical trials with a 1.6-year follow-up have also shown that left ventricular dysfunction is an independent predictor of stroke while LA size is not (23). In these studies, the follow-up periods were short, and the relationship between CHA_2_DS_2_-VASc score and LA size was not addressed. In the present study, with the longest follow-up of AF patients, we showed that LA size was positively and linearly correlated with CHA_2_DS_2_-VASc score but did not provide additional information in the risk stratification of stroke.

### Clinical Implications of Positive Correlation Between LA Size and CHA_2_DS_2_-VASc Score

LA enlargement could promote blood stasis, which in turn predisposes thrombus formation, especially in the left atrial appendage (24). Many studies have shown that LA dimension could reflect the AF burden, which has been reported to be associated with the risk of stroke in several device-detected AF trials (25–27). Therefore, since AF burden is difficult to measure in patients without cardiac implanted electronic devices, it has been proposed that LA dimension could be used as a good surrogate marker of AF burden and duration. However, while measurement of LA dimension may add additional information in AF patient care, echocardiography is a less feasible modality and not recommended for broad screening of unselected populations in the current guidelines and trials (28, 29). Our study first showed the positive correlation between LA size and the CHA_2_DS_2_-VASc score. This result further increases the simplicity of quantification of AF burden and duration for screening a large number of AF patients. Application of the CHA_2_DS_2_-VASc score seems useful in assessing the AF burden and duration in large AF population screen without the need to perform echocardiography or implantation of cardiac electronic device.

Initially, we expected that echocardiographic LA size was a good surrogate measurement of AF burden and LA size was the ultimate independent predictor of stroke risk for patients with AF. However, echocardiography may not be immediately available in all clinics. Calculation of CHA_2_DS_2_-VASc score is an easier tool to use in clinics to predict stroke risk for patients with AF, and we hypothesized that CHA_2_DS_2_-VASc score was effective to predict stroke risk through its correlation with LA size. However, unexpectedly, we found that CHA_2_DS_2_-VASc score, but not LA size, was the only independent predictor of AF stroke risk.

### CHA_2_DS_2_-VASc Score Is More Important Than LA Size in AF Stroke Risk Assessment

LA size is a surrogate measurement of AF burden, but whether AF burden is directly related to AF stroke risk remains debated (30–32). A significant proportion of cause of stroke in AF patients may be atheroemboli or atherosclerotic occlusion from carotid arteries, aorta, or other sources. This atheroembolic burden is related to atherosclerosis burden. Interestingly, all of the components of CHA_2_DS_2_-VASc score are risk factors of systemic atherosclerosis. Our results imply the possibility that a large proportion of cause of stroke in AF patients is atheroemboli or atherosclerotic occlusion from the arterial circulating system. To sum up, application of the CHA_2_DS_2_-VASc score still remain the mainstream to assess the stroke risk in large AF populations.

In addition to the possibility that the cause of stroke in AF patients is atheroemboli or atherosclerotic occlusion from the arterial circulating system, another possibility is that the cause of stroke is the thrombus in the left atrial appendage and the mechanism of thrombus formation in the left atrial appendage is a pathological process in the appendage endocardium, which is similar to vascular endothelial dysfunction or an atherosclerosis process, but not enlarged LA or left atrial appendage. It is logical to speculate that risk factors of endothelial dysfunction or atherosclerosis, such as the components of CHA_2_DS_2_-VASc score, are also risk factors of appendage endocardial pathology.

Our study first shows a positive correlation between CHA_2_DS_2_-VASc score and LA size. Regarding the plausible explanation of this correlation, all of the components of CHA_2_DS_2_-VASc score are predisposing factors of AF attack and LA remodeling. Patients with a higher CHA_2_DS_2_-VASc score may have more predisposing factors of AF attack and suffer from more AF episodes, which may increase their LA size.

### Limitations

The major limitation of the present study is no measurement of the LA volume. Because this is a large cohort with standard cares for AF patients, LA volume is not a routine measurement in our cohort. LA diameter measurement is more widely employed in daily practice. Furthermore, it has been shown that LA volume can be well-estimated from LA diameter using a non-linear equation with an elliptical model (33). Finally, a recent large-scale AF follow-up study also used echocardiographic M-mode measurement of the anteroposterior diameter of LA in parasternal long-axis view but not LA volume as one of their study parameters (11).

## Conclusion

In conclusion, the present study shows a positive correlation between echocardiographic LA size and CHA_2_DS_2_-VASc score. LA size is not an independent predictor of AF-related stroke but provides a diagnostic value to predict AF-related stroke risk through its association with CHA_2_DS_2_-VASc score. Calculation of the CHA_2_DS_2_-VASc score may be an alternative to measure echocardiographic LA size when evaluating the risk of stroke for patients with AF.

## Data Availability Statement

The original contributions presented in the study are included in the article/supplementary material, further inquiries can be directed to the corresponding author/s. The corresponding author has full access to all of the data in the study and takes responsibility for the integrity of the data and the accuracy of the data analysis.

## Ethics Statement

The studies involving human participants were reviewed and approved by National Taiwan University Hospital. The patients/participants provided their written informed consent to participate in this study.

## Author's Note

The content of this manuscript has been presented at the ESC Congress 2019.

## Author Contributions

C-FT and P-SH: study design, analyses, conducting, and manuscript preparation. J-JC, S-NC, and L-PL: study design. F-CC: analyses. T-TL: study design and analyse. J-JH: study design and planning. C-TT: study design, analyses, and manuscript preparation. All authors contributed to the article and approved the submitted version.

## Conflict of Interest

The authors declare that the research was conducted in the absence of any commercial or financial relationships that could be construed as a potential conflict of interest.

## Abbreviations

AF: atrial fibrillation
AUC: area under curve
DM: diabetes mellitus
ECG: electrocardiography
LA: left atrium
LV: left ventricle
NTUAFR: National Taiwan University Atrial Fibrillation Registry
ROC: receiver operator characteristics
TIA: transient ischemic attack.
